# LIFE COURSE APPROACH TO CAREGIVING FOR AGING PARENTS: THE ROLE OF ADVERSE CHILDHOOD EXPERIENCES

**DOI:** 10.1093/geroni/igae098.3378

**Published:** 2024-12-31

**Authors:** Jooyoung Kong, Stephanie Robert, Jason Fletcher

**Affiliations:** University of Wisconsin Madison, Madison, Wisconsin, United States; University of Wisconsin Madison, Madison, Wisconsin, United States; University of Wisconsin Madison, Madison, Wisconsin, United States

## Abstract

The high strain and burden of caring for aging parents have been well-documented. Few but growing studies have found the effects of earlier distal factors, such as childhood maltreatment, on negative mental health for caregivers of aging parents. Taking a life course approach to intergenerational caregiving, this study examined the long-term impact of adverse childhood experiences (ACEs) on adult caregivers of aging parents. Using data from the Midlife in the United States (MIDUS), we analyzed a sample of 412 caregivers of aging mothers. We conducted a series of latent class analyses (LCA) for a holistic assessment of the patterns of ACEs among the caregivers. Respondents’ exposure to ACEs available in MIDUS included parental neglect, emotional abuse, physical abuse, sexual assault (perpetrator not specified), living with a household member with a substance use issue, and parental divorce/separation/death during childhood. Among the caregivers of mothers, we identified a five-latent-class structure: No/low ACEs (60%), Neglect and Household Challenges (12%), Paternal Abuse (5%), Parental Physical Abuse (16%), and Substantial ACEs (7%). The respondent’s education was significantly associated with latent class membership. Caregivers in the Substantial ACEs class showed consistently worse physical and mental health than those in the No/low ACEs class. The current study contributes to raising awareness of adult-child caregivers who experienced childhood trauma, who may deal with accumulated disadvantages from childhood adversities, their potential correlates, and current caregiving. Further investigations will help find ways to reduce ACEs’ impact in order to promote the health of these caregivers and strengthen their well-being.

